# Evolving gene banks: improving diverse populations of crop and exotic germplasm with optimal contribution selection

**DOI:** 10.1093/jxb/erw406

**Published:** 2016-11-10

**Authors:** W A Cowling, L Li, K H M Siddique, M Henryon, P Berg, R G Banks, B P Kinghorn

**Affiliations:** 1 The UWA Institute of Agriculture M082, The University of Western Australia, Stirling Highway, Perth WA, Australia; 2 Animal Genetics and Breeding Unit, University of New England, Armidale NSW, Australia; 3 SEGES, Pig Research Centre, Axeltorv, Copenhagen V, Denmark; 4 School of Animal Biology, The University of Western Australia, Stirling Highway, Perth WA, Australia; 5 NordGen, Nordic Genetic Resource Center, Postboks, NO-1431 Ås, Norway; 6 School of Environmental & Rural Science, University of New England, Armidale NSW, Australia

**Keywords:** Crop breeding, effective population size, evolving gene bank, mean population coancestry, optimal contribution selection, pre-breeding, self-pollinating crops

## Abstract

We simulated pre-breeding in evolving gene banks – populations of exotic and crop types undergoing optimal contribution selection for long-term genetic gain and management of population genetic diversity. The founder population was based on crosses between elite crop varieties and exotic lines of field pea (*Pisum sativum*) from the primary genepool, and was subjected to 30 cycles of recurrent selection for an economic index composed of four traits with low heritability: black spot resistance, flowering time and stem strength (measured on single plants), and grain yield (measured on whole plots). We compared a small population with low selection pressure, a large population with high selection pressure, and a large population with moderate selection pressure. Single seed descent was compared with S_0_-derived recurrent selection. Optimal contribution selection achieved higher index and lower population coancestry than truncation selection, which reached a plateau in index improvement after 40 years in the large population with high selection pressure. With optimal contribution selection, index doubled in 38 years in the small population with low selection pressure and 27–28 years in the large population with moderate selection pressure. Single seed descent increased the rate of improvement in index per cycle but also increased cycle time.

## Teaser

For the first time in self-pollinating crops, we apply optimal contribution selection to manage long-term genetic gain and genetic diversity in pre-breeding populations known as evolving gene banks.

## Introduction

Large genetic diversity exists in wild and landrace relatives of crop plants, but most of this diversity is held in gene banks and not in active breeding programmes. Gene banks are repositories for wild and landrace types from a crop’s primary, secondary or tertiary gene pool (Harlan and de Wet, 1971). Serious measures have been taken to improve long-term survival of seed in crop gene banks. The Svalbard Global Seed Vault project (http://www.nordgen.org/sgsv/) is located in the permafrost 1300 km north of the Arctic Circle, and is the world’s largest secure seed storage. Seed of many of the world’s legume crops are stored in this and other gene banks (Foyer *et al.*, 2016). The question that often arises is: how best to use these global genetic resources to improve crop breeding and crop production?

Many crop breeding programmes have narrow genetic diversity (Cowling, 2013), especially grain legumes (Singh *et al.*, 2014). Genetic resources, especially wild or landrace lines from the primary gene pool, will play an important role in future crop improvement especially with the help of molecular genetic technologies (Tester and Langridge, 2010). ‘Advanced backcross QTL’ with whole-genome markers aids the incorporation of useful quantitative alleles into elite breeding programmes (Tanksley and Nelson, 1996). Allelic variation can be identified in gene bank collections for key phenotypic traits such as flowering time (Keilwagen *et al.*, 2014). ‘Exotic genetic libraries’ were proposed to enhance genetic diversity available to breeders (Zamir, 2001). Breeding efforts to improve the yield, disease resistance and quality of several grain legumes are constrained by a low level of genetic diversity in breeding programmes (Croser *et al.*, 2003; Singh *et al.*, 2014). Genetic resources have improved the range of adaptation, disease resistance and quality of many grain legume species, but the question remains: how best to exploit these resources in the future (Siddique *et al.*, 2013)?

Plant breeders are reluctant to cross outside of elite pools, because migration of potentially valuable alleles from exotic germplasm into elite breeding pools is usually accompanied by a decrease in economic performance (Rasmusson and Phillips, 1997). Controlled backcrossing can be used to manage the migration of positive alleles from wild to domestic populations while reselecting for domestication traits (Cowling *et al.*, 2009). However, it is not certain that valuable donor alleles, especially those with quantitative effects, will survive the backcrossing and selfing process as a result of linkage disequilibrium, small population size and genetic drift (Cowling, 2013). Donor alleles are subject to interactions with the genetic background of the recurrent parent, which may result in unsuccessful outcomes of backcrossing (Hospital, 2005). Negative donor alleles may be linked to the target donor alleles, leading to ‘linkage drag’ and lower commercial performance than the recurrent variety (Hospital, 2005). This problem is exacerbated by the large linkage blocks found in elite breeding programmes of self-pollinating crops, which are 200 times larger than in out-crossing species such as maize (Rostoks *et al.*, 2006). Rapid cycles of recurrent selection will increase the frequency of effective recombination compared with backcrossing, and this will help to break up linkage blocks and reduce linkage drag.

To help make exotic alleles available to breeders and avoid extensive backcrossing, we propose active pre-breeding in ‘evolving gene banks’ – populations of exotic and crop types undergoing optimal contribution selection (OCS) for long-term genetic gain and retention of population genetic diversity. The evolving gene bank is based on the ‘animal model’ of breeding, which exploits information from relatives to estimate breeding values of each related individual in the pedigree (Lynch and Walsh, 1998). In the animal model, the accuracy of predicted breeding values is increased through the use of relationship information, normally from pedigrees, but potentially also from ‘realised’ (genomic) relationship information (Hayes *et al.*, 2009). A version of the animal model for self-pollinating crops included both crossing and selfing relationships in the pedigree, and resulted in high accuracy of prediction and potentially high rates of genetic improvement in S_0_-derived recurrent selection for a low heritability trait in *Pisum sativum* L., field pea (Cowling *et al.*, 2015).

The success of evolving gene banks is highly dependent on the method of selection. Truncation selection is predicted to maximize genetic merit in the offspring generation. However, this is generally not the best strategy for maximizing long-term genetic gain, because the highest-ranked individuals tend to be closely related. Lack of attention to genetic diversity will generally lead to reduced opportunity for genetic gains in later generations.

OCS provides a potential solution to this problem. OCS aims to increase the rate of genetic gain in a breeding population for a given rate of inbreeding by optimizing the genetic contribution of each individual to the next generation (Henryon *et al*., 2014, 2015; Woolliams *et al.*, 2015). Selection is based not only on high genetic merit, but also on weighted-average relationship of the selected individuals. This can be done in a manner that maximizes next-generation genetic gains for a nominated parental coancestry (Meuwissen, 1997; Grundy *et al.*, 1998), or it can involve a nominated balance between next-generation genetic gains and parental coancestry (Kinghorn *et al.*, 2002; Kinghorn, 2011). In addition to animal breeding, OCS has been applied to out-crossing forest tree species (Hallander and Waldmann, 2009; Kerr *et al.*, 2015), but not yet to self-pollinated crop species. Useful improvements in OCS may be possible through addition of genomic relationship information (Woolliams *et al.*, 2015).

In OCS, there is no simple method to calculate the relative emphasis to place on genetic gain versus genetic diversity in order to maximize genetic merit at a given future generation. However, this can be estimated using stochastic simulation, as we have done in this paper.

We modelled recurrent selection for an economic index beginning from a hypothetical base population made up of crosses between exotic lines from the primary gene pool (wild or landrace types) and elite crop varieties. The selection index included traits in field pea with known or estimated heritability and genetic and phenotypic correlations on a single plant basis (Beeck, 2005; Beeck *et al*., 2008*a*, *b*; Cowling *et al.*, 2015). We included plot grain yield in the index, which extended selection cycles by one year. For the first time in self-pollinating crops, we applied OCS to improve long-term genetic progress and manage population coancestry, and compared OCS to truncation selection with random or assortative mating. We compared small and large pre-breeding populations, various selection intensities, and various levels of selfing within each cycle of selection. We explored the utility of OCS to improve long-term genetic gain and manage genetic diversity in evolving gene banks.

## Materials and methods

### Population size

Three population types were compared in these studies: (i) a small population with 250 progeny per cycle and low selection pressure of 50 matings per cycle and 5 progeny per mating (small-low); (ii) a large population with 1000 progeny per cycle and high selection pressure of 20 matings per cycle and 50 progeny per mating (large-high); and (iii) a large population with 1000 progeny per cycle and moderate selection pressure of 50 matings per cycle and 20 progeny per mating (large-moderate). These populations represent a range of breeding costs from low budget (small-low) to high budget (large-high and large-moderate), and are feasible scenarios for evolving gene banks in a range of contexts for crop pre-breeding.

For the small-low and large-moderate populations, a founder population of 100 individuals was generated (see ‘Simulation of individual plants’, below) and ranked on economic selection index. One-half of the founders were randomly assigned to elite status and one-half were assigned to exotic status. Random mating was then simulated between elite and exotic founders, always using one elite and one exotic parent in each mating and using each founder parent once only, to begin the recurrent selection process (see ‘Simulation of individual plants’, below). For the large-high population, the founder population was reduced to 40 individuals and 20 matings. The cross progeny are designated S_0_ progeny following the nomenclature for segregating cross progeny from heterozygous parent plants; the F_2_ generation is equivalent to S_0_ when the parents are inbred (Bernardo, 2010).

### Economic selection index

**Fig. 1.**
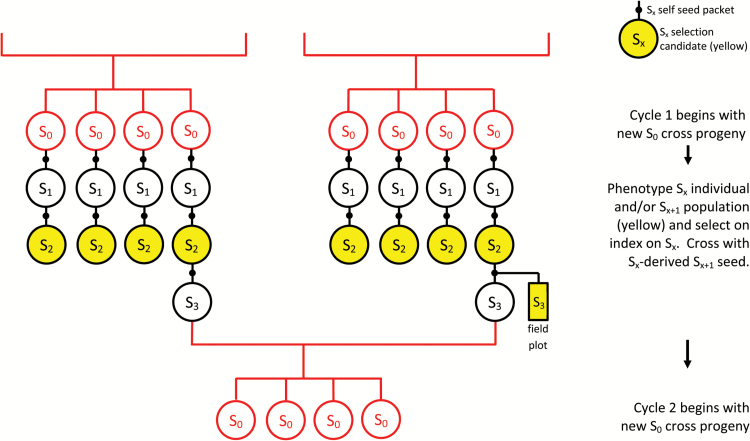
Generalized model for index selection in annual crop breeding, where selfing (black lines) occurs within cycles to produce self progeny (black circles). Crossing (red lines) and cross progeny (red circles) signify the beginning of the next cycle. In this example, S_2_ individuals and their S_3_ populations (marked in yellow) are phenotyped for target traits, and index selection occurs on the S_2_. S_2_-derived S_3_ seed are used in crossing. In general, phenotyping occurs on S_x_ individuals and/or S_x_-derived S_x+1_ plots (yellow) and index selection occurs on the S_x_.

Four traits in field pea contributed to the economic selection index: three can be measured on a single plant basis (black spot resistance, stem strength and flowering time) and one is measured on a plot basis (grain yield). We assumed knowledge of the starting mean values, standard deviations and narrow-sense heritability for these traits in the base population according to results from previous research (Beeck, 2005; Beeck *et al*., 2008*a*, *b*; Cowling *et al.*, 2015) and our best estimates (Table 1). In our simulated BLUP analysis, true breeding values were generated for every plant in the pedigree including selfs as described below. Phenotypes were measured on single S_x_ plants or on S_x_-derived S_x+1_ plots (Fig. 1). For grain yield, we used records from S_x_-derived S_x+1_ plots to predict breeding values of S_x_ individuals (Bernardo, 2010; Walsh and Lynch, 2016*b*). We assumed initial values for all traits on S_x_ plants (Table 1). The genetic and phenotypic correlations between traits were also based on published information or best available knowledge (Table 2).

**Table 1.** Starting values for mean, standard deviation and narrow-sense heritability in the base population for four traits, and economic weight for each trait used to calculate the selection indexThe emphasis placed on each trait was calculated using the desired gains approach (Brascamp, 1984), implemented using the program Desire (Kinghorn, 2016*b* ). The index weight was used for calculating the phenotypic index. For each trait except flowering time, the selection goal was for increasing positive values. For flowering time, the selection goal was neutral – to maintain the average over time. For blackspot resistance, the starting value (100%) represents the average level of disease in the base population (increases in resistance through selection will increase this value above 100% over time).

Trait	Unit	Starting value	Standard deviation	Narrow-sense heritability	Economic weight	Index weight	Selection goal
Blackspot resistance	%	100	20	0.3	0.1409	0.0403	increase
Stem strength	mm	1.2	0.3	0.3	4.124	1.115	increase
Flowering time	days	80	20	0.5	-0.02	-0.005	neutral
Grain yield	t ha^-1^	1.5	0.25	0.3	4.265	1.616	increase

**Table 2.** Estimated genetic and phenotypic correlations between traits applied to the base populationPhenotypic correlations are above the diagonal; genetic correlations are below.

Trait	Blackspot resistance	Stem strength	Flowering time	Grain yield
Blackspot resistance	-	−0.05	0.25	0.15
Stem strength	−0.1	-	0.0	0.1
Flowering time	0.3	0.0	-	−0.1
Grain yield	0.2	0.15	−0.15	-

The emphasis placed on each trait was calculated using the desired gains approach (Brascamp, 1984), implemented using the program Desire (Kinghorn, 2016*b*). This involved specifying the desired relative responses to selection on phenotypic index (not exploiting information from relatives) for the traits involved, but with constraint to adhere to the response surface of all possible outcomes. The result involved both the index weights *b* that were predicted to give the outcome specified, plus the implied economic weightings *e* that result in index weightings *b* when applying classic selection index theory (Brascamp, 1984; Kinghorn, 2016*b*). These economic weightings (Table 1) were adopted for calculation of BLUP-based economic index values, although in fact the pattern of response across traits could deviate from that desired because of use of information from relatives. The index weight was used for calculating the phenotypic index (Table 1).

### Generation interval vs cycle time in breeding annual self-pollinating crops

The term ‘generation interval’ from animal breeding must be used cautiously in annual self-pollinating crops, because several generations of selfing may occur within each cycle of recurrent selection. In this paper, the term ‘cycle time’ refers to the duration of each cycle of recurrent selection, and ‘generations’ refers to selfing generations within cycles. We modelled selfing crop breeding with 0, 3 or 5 generations of single seed descent within cycles, resulting in cycle times of 2, 3 and 4 years and 30, 20 and 15 cycles over 60 years. The S_x+1_ generation (Fig. 1) in each cycle was devoted to a field trial for measuring yield (Table 3). In order to calculate cycle time in years, we assume that each selfing generation during single seed descent can be completed in 4 months and that sufficient self seed can be harvested from a single S_x_ plant to sow a plot of S_x+1_ seed for yield measurement in the following year (Table 3). Models were compared for the small-low, large-high, and large-moderate population.

**Table 3.** Cycle times in the plant model with 0, 3, and 5 generations of single seed descent within cyclesBlack spot resistance, stem strength, and flowering time were recorded on single pea plants in the next-to-final generation within each cycle, and grain yield was recorded the following year in field plots sown with self seed harvested from these plants.

S_gen_	Year 1	Year 2	Year 3	Year 4	Year 5
0	Cycle 1 starts:	Field trial S_0_-	Cycle 2 starts:	Field trial S_0_-	Cycle 3 starts:
	Cross | S_0_ |	derived S_1_ bulks	Cross | S_0_ |	derived S_1_ bulks	Cross | S_0_ |
3	Cycle 1 starts:	S_1_ | S_2_ | S_3_ |	Field trial S_3_-	Cycle 2 starts:	S_1_ | S_2_ | S_3_ |
	Cross | S_0_ |		derived S_4_ bulks	Cross | S_0_ |	
5	Cycle 1 starts:	S_1_ | S_2_ | S_3_ |	S_4_ | S_5_ |	Field trial S_5_-	Cycle 2 starts:
	Cross | S_0_ |			derived S_6_ bulks	Cross | S_0_ |

### Selection criteria

Two criteria were available on individuals to make selection decisions for individual i:

Phenotypic index:

$$Pindex_{i}=\;\overset{nTraits}{\underset{j=1}{\sum }}b_{j}.P_{i,j}$$

where *b* is a vector of selection index weights, as described above, and $P_{i,j}$is the phenotype of individual *i* for trait j.

Best linear unbiased prediction (BLUP)-estimated breeding value index:

$$BLUPindex_{i}=\overset{nTraits}{\underset{j=1}{\sum }}e_{j}.EBV_{i,j}$$

where *e* is a vector of implied economic weights, as described above, and $EBV_{i,j}$ is a vector of estimated breeding values for individual *i* calculated by BLUP.

As shown in the formulae above, $Pindex_{i}$ was calculated from the phenotypes of the four traits, and $BLUPindex_{i}$ was calculated from estimated breeding values based on multiple-trait BLUP analysis on phenotypes and pedigree information generated in the simulations. Modelling of all traits was based on the starting values and genetic parameters in Tables 1 and 2.

### Simulation of individual plants

Simulations were carried out using the PopSim module of Genup (Kinghorn, 2016*a*), which was developed and used in the context of the animal model (Webb *et al.*, 2012). PopSim was modified to include OCS (Matesel) as an option for mate selection and mate allocation decisions at each breeding cycle, following the approach of Kremer *et al.* (2010). PopSim was modified to handle bisexuality and selfing as required for self-pollinating crops.

Breeding values can be estimated for S_x_ individuals provided they have measured relatives in the analysis (Walsh and Lynch, 2016*a*). Phenotypes were measured on single S_x_ plants or on S_x_-derived S_x+1_ plots (Fig. 1). Under certain conditions, records from the S_x_-derived S_x+1_ generation may be used to predict genetic values of S_x_ individuals (Bernardo, 2010; Walsh and Lynch, 2016*b*). Once the S_x_ plants were selected on the basis of index, then S_x+1_ remnant seeds were used in crossing (Fig. 1).

For selfing that occurred before phenotyping, PopSim generated a single random self progeny from each plant to start the next selfing generation. This is equivalent to single seed descent (Allard, 1960). The number of generations of selfing before phenotyping and selection was defined as S_gen_ and we compared simulations with S_gen_=0, 1, 2, 3, 4 and 5. In annual self-pollinating crops, selfing normally occurs after selection and before crossing, and therefore remnant self seed must be used in crossing. Selfing after selection of S_x_ individuals, in order to obtain seed for crossing, was defined as S_sel_ and we compared simulations with S_sel_=0 and 1. We also simulated the typical case in animals, where S_gen_=0 and S_sel_=0.

In Popsim, a foundation population is simulated with phenotypes generated as follows:

$$P_{i,j}=X_{j}+A_{i,j}+E_{i,j}$$

where

$$A_{i,j}=RanA_{i,j}.\sigma_{A_{j}}$$

$$E_{i,j}=RanE_{i,j}.\sigma_{E_{j}}$$

and$\;P_{i,j}$ is phenotype of individual *i* for trait *j*; $X_{j}$ is initial population mean for trait *j*;$\;\sigma_{A_{j}}$ is the standard deviation among additive genetic values of individuals for trait *j* which equals the square root of (narrow-sense heritability times population phenotypic variance);$\;RanA_{i,j}$ is the $j^{th}$ element of a vector of normal deviates that are correlated within individuals $\left(RanA_{i,.}\right)$ by the additive genetic correlations among the traits involved;$\;\sigma_{E_{j}}$ is the standard deviation among environmental deviations of individuals for trait *j* which equals the square root of (1 minus narrow-sense heritability times population phenotypic variance);$\;RanE_{i,j}$ is the $j^{th}$ element of a vector of normal deviates that are correlated within individuals $\left(RanE_{i,.}\right)$ by the environmental correlations among the traits involved. We ignored fixed effects, such as sex, season, known QTL and common environment, which were assumed to be zero.

The foundation (base) population was generated in the first breeding cycle. The numbers of individuals generated complies with user settings for initial breeding population size.

For each subsequent breeding cycle, phenotypes of progeny were generated as follows [in crossing, each plant was used as either a male (sire) or female (dam), and in selfing, a plant was used both as a sire and a dam]:

$$\begin{array}{l} P_{i,j}=\frac{A_{Sire(i),j}+A_{Dam(i),j}}{2}\\ \;+RanA_{i,j}.\;\sqrt{\frac{2-F\left(Sire\left(i)\right)-F(Dam(i)\right)}{4}}.\sigma_{A_{j}}+RanE_{i,j}.\sigma_{E_{j}} \end{array}$$

where $F_{i}$ is the inbreeding coefficient of individual i.

The average and standard deviation of index and population coancestry was recorded for ten replicate runs per cycle over 30 cycles for each model in PopSim (See Supplementary Table S1 at *JXB* online). In the first year, crossing occurred among exotic and elite varieties to generate the base population, after which recurrent selection began. Therefore cycle 31 represents 30 cycles of recurrent selection (Supplementary Table S1).

### Selection strategy and mating designs

The standard method was truncation selection based on index, followed by random or assortative mating among the selected parents. In truncation selection, each selected parent was used only once in crossing. For the small-low and large-moderate population, the top 100 parents for index were selected to generate 50 matings per cycle. For the large-high population, the top 40 parents for index were selected to generate 20 matings per cycle. We also compared truncation selection based on $BLUPindex_{i}$ (an index of estimated breeding values) with truncation selection based on $Pindex_{i}$ (an index of phenotypic values).

**Fig. 2.**
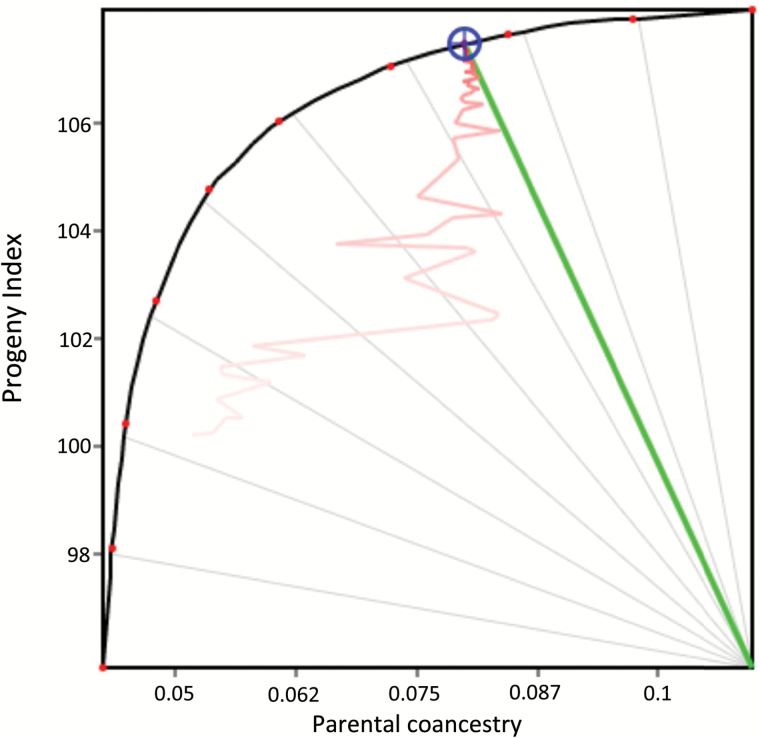
A screenshot of a response frontier as displayed in Matesel. The curve is the frontier of optimal contributions, where each point on the frontier represents an optimal mating list for the corresponding relative emphasis on progeny index and parental coancestry. The top-right of the frontier is 0 degrees, with full emphasis on progeny index, and the bottom-left is 90 degrees, with full emphasis on lowered parental coancestry. The solution has settled on the frontier at the 25 degree ‘Target Degree’ line.

For the first time in a self-pollinating crop, we used optimal contribution selection (OCS) to manage long-term genetic gain and genetic diversity, and compared this with truncation selection. OCS was based on the breeding programme implementation platform ‘Matesel’ (Kinghorn and Kinghorn, 2016). Matesel dictates which individuals to select and the actual mating allocations and/or selfings to be made. Graphical representation of key outcomes aids the user to dynamically edit the objective function, and thus steer the outcome to a solution that meets the breeding goal (Fig. 2).

We edited the objective function in Matesel to achieve different outcomes. This involved changing the balance between progeny index (reflecting genetic gain) and mean parental coancestry. The point at the top-right of Fig. 2 represents the mating list that maximizes genetic gain under any constraints specified. This will involve selection of the fewest possible best plants with no regard to genetic diversity. The point at the bottom-left of the graph represents the mating list that maximizes genetic diversity. This will involve selection of many plants, but with higher contributions from those that are less related to the rest of the material selected. The curved frontier shows the range of optimal solutions across all levels of balance between these two key factors. In this case the balance strategy is specified on a scale from 0 degrees (top-right) to 90 degrees (bottom-left), and the solution shown is for 25 target degrees.

Selected parents were used either once only in crossing, which was directly comparable to truncation selection in terms of number of unique individuals in crossing, or used up to a maximum of five matings (but within the same cycle), as determined by OCS, which was a more aggressive approach to allow stronger improvements in index.

The balance between high selection intensity and low population coancestry was varied by using target 45 degrees (to emphasise improvement in index) and target 60 degrees (to emphasise low population coancestry).

### Population inbreeding and coancestry

As PopSim progressed through simulation of each cycle of recurrent selection, it calculated the mean population index and population coancestry at the end of each cycle. Parental coancestry is directly related to genetic diversity, effective population size and the rate of inbreeding (Kinghorn *et al.*, 2008). The rate of inbreeding (ΔF) is related to the inverse of effective population size $(N_{e}):$

$$\Delta F=\frac{1}{2N_{e}}$$

In an ideal population,

$$\frac{1}{2N_{e}}=\frac{x\text{'}x}{2}$$

where *x* is a column vector of relative contributions of candidate individuals, summing to unity. For example, for four unrelated parents used equally:

$$\frac{1}{2N_{e}}=\frac{x'x}{2}=\frac{1}{8}$$

Mean parental coancestry (f) is $\frac{x'Ax}{2}$ where *A* is the numerator relationship matrix among candidate individuals. Parental coancestry thus improves on $\frac{x'x}{2}$ for managing inbreeding rate, and hence genetic diversity, as it accounts for the relationship between individuals.

We use mean parental coancestry (f) as a measure of genetic diversity in the population during recurrent selection. High population coancestry (approaching 1) indicates that most population diversity has been utilised for improvements in index. In graphical presentations, we use ‘1 − coancestry’ (1−f) to view the amount of genetic diversity remaining in the population.

## Results

### Comparison of the animal model with the plant model, with various levels of selfing

For the small-low population, the economic index was ~23.8 in the base population (Supplementary Table S1).

**Fig. 3.**
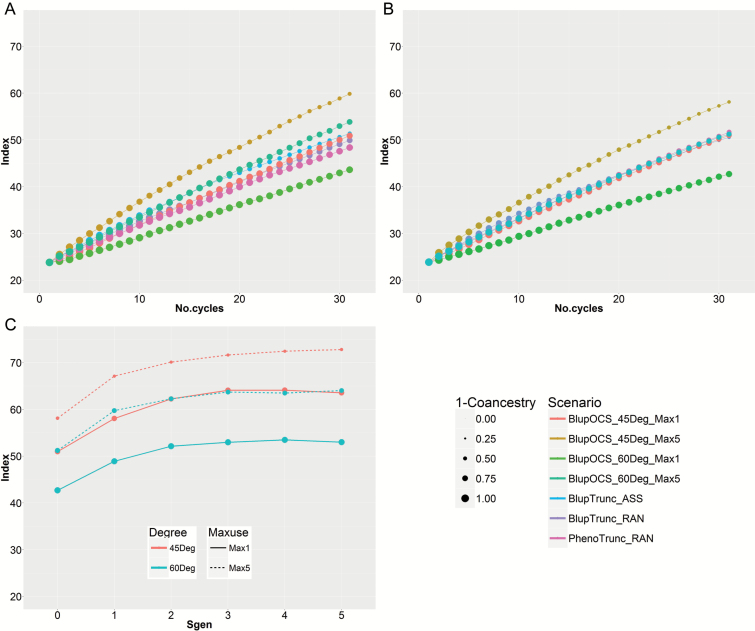
Modelling of 30 cycles of S_0_-derived recurrent selection in the small population based on (A) the animal model with S_sel_=0 and S_gen_=0, (B) the plant model with S_sel_=1 and S_gen_=0, and (C) index achieved at 30 cycles in the plant model for Sgen=0, 1, 2, 3, 4 and 5. Size of dots is in linear proportion to 1 – population coancestry (‘1−Coancestry’). ‘BlupTrunc’, truncation selection based on index; ‘PhenoTrunc’, truncation selection based on phenotypic values; ‘RAN’, random mating; ‘ASS’, assortative mating; ‘BlupOCS’, index-based optimal contribution selection; ‘Degree’, OCS parameter which changes emphasis on index or coancestry; ‘45Deg’, OCS option 45 degrees which favours index; ‘60Deg’, OCS option 60 degrees which favours low population coancestry; ‘Maxuse’, OCS option which limits the maximum use of parents; ‘Max1’, OCS option for maximum single use of each parent; ‘Max5’, OCS option for maximum five uses of each parent.

The normal situation in animals, with S_sel_=0 and S_gen_=0, showed the following outcomes after 30 cycles of recurrent selection (Fig. 3A):

(i) Truncation selection with assortative mating (BlupTrunc_ASS) achieved slightly higher index (51.4) than with random mating (49.9) (BlupTrunc_RAN), but with much higher population coancestry (coancestry increased from 0.24 with random mating to 0.48 with assortative mating). Truncation selection based on phenotypic values (PhenoTrunc_RAN) achieved lower index (48.4) but also lower population coancestry (0.15).
(ii) OCS with emphasis on low coancestry (target 60 degrees) and maxuse=1 (BlupOCS_60Deg_Max1) resulted in the lowest population coancestry (0.13) but also the lowest gain in index (43.6) after 30 cycles. This scenario conserved the most genetic diversity in the population.
(iii) OCS achieved the highest index (59.8) with emphasis on high index (target 45 degrees) and maxuse=5 (BlupOCS_45Deg_Max5), with similar population coancestry (0.50) to truncation selection with assortative mating (BlupTrunc_ASS).
(iv) OCS achieved a reasonable compromise for conservation of genetic diversity when emphasis was on low coancestry (BlupOCS_60Deg_Max5), with index 53.8 and population coancestry 0.26 after 30 cycles.

In the plant model based on recurrent selection in *P. sativum* (Cowling *et al.* 2015) with S_sel_=1 and S_gen_=0 (Fig. 3B), the model shows similar results to the situation in animals, where S_sel_=0 and S_gen_=0 (Fig. 3A), but with an increase in population coancestry of ~0.08 and a small decrease in index (1 to 2 index units) after 30 cycles. The highest index (58.1) was achieved by OCS with emphasis on high index (BlupOCS_45Deg_Max5), but with population coancestry 0.57. Once again, a reasonable compromise for genetic resource conservation was obtained by OCS with emphasis on low coancestry (BlupOCS_60Deg_Max5), with index 51.2 and population coancestry 0.33 after 30 cycles of recurrent selection.

The effect of selfing with S_gen_=0, 1, 2, 3, 4 and 5 on index and coancestry at the end of 30 cycles of recurrent selection is shown in Fig. 3C. Under all scenarios, there was a large increase (8–9 units) in economic index with one generation of selfing (S_gen_=1), and lower incremental increases with further selfing. For OCS with emphasis on high index (BlupOCS_45Deg_Max5), index values increased from 58.1 (S_gen_=0), 67.1 (S_gen_=1), 71.6 (S_gen_=3) to 72.8 (S_gen_=5). Population coancestry increased from 0.57 (S_gen_=0), 0.61 (S_gen_=1) to 0.62 (S_gen_=3) and then reduced slightly to 0.60 (S_gen_=5). The same pattern occurred in all OCS scenarios, sometimes with a small drop in index at S_gen_=5 (Fig. 3C).

### Comparison of S_0_-derived with S_3_-derived and S_5_-derived recurrent selection in the small population

**Fig. 4.**
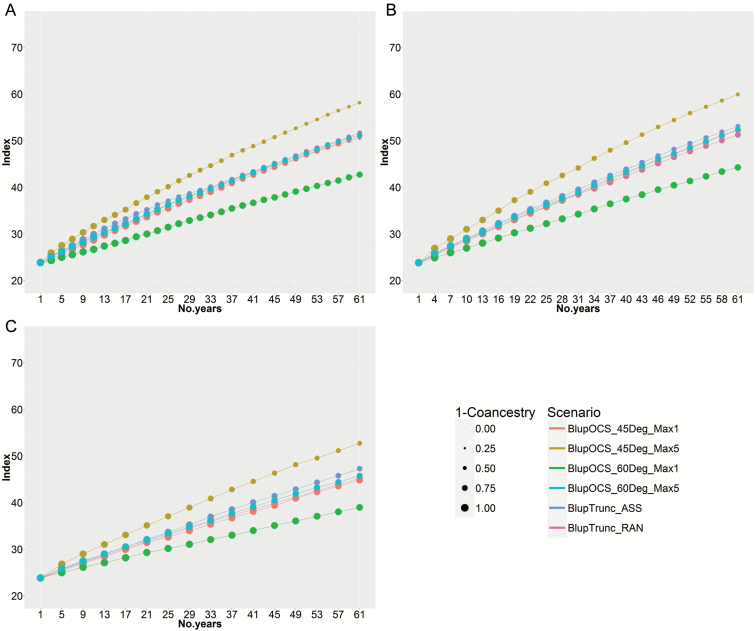
Modelling of 30 cycles of S_0_-derived recurrent selection in the small population based on (A) the plant model with S_sel_=1 and S_gen_=0, (B) three generations of single seed descent to S_3_, and (C) five generations of single seed descent to S_5_. Size of dots is in linear proportion to 1 – population coancestry. For abbreviations, see Fig. 3 legend.

In the small-low population with S_gen_=0 and S_sel_=1, the index doubled in 38 years with population coancestry 0.41 in the high-index OCS option (BlupOCS_45Deg_Max5) (Fig. 4A). In contrast, doubling of index for truncation selection with random mating took 52 years (population coancestry 0.34), and in the OCS solution which emphasized coancestry (BlupOCS_60Deg_Max5), doubling time took 52 years with population coancestry 0.29.

There was a higher index achieved per cycle with selfing to S_gen_=3 (Fig. 4B), but this advantage was reduced by the longer (3-year) cycles, with doubling of index in 36 years and population coancestry 0.34 in the high-index OCS option (BlupOCS_45Deg_Max5). In the OCS solution favouring coancestry (BlupOCS_60Deg_Max5), doubling time took 50 years with population coancestry 0.25.

There was no benefit from continuing single seed descent to S_gen_=5 (Fig. 4C), with doubling of index in 48 years and population coancestry 0.33 in the high-index OCS option (BlupOCS_45Deg_Max5).

### Comparison of S_0_-derived with S_3_-derived and S_5_-derived recurrent selection in the large population with high selection pressure

**Fig. 5.**
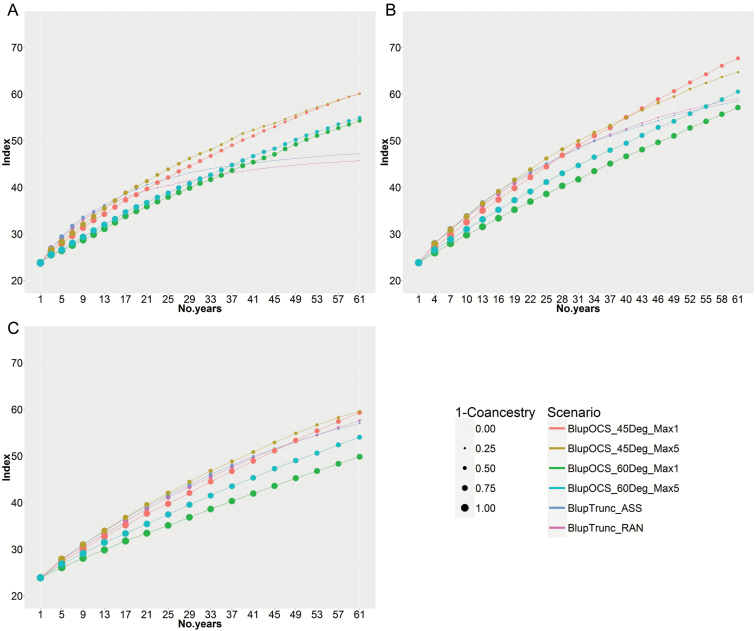
Modelling of 30 cycles of S_0_-derived recurrent selection in the large population with high selection pressure based on (A) the plant model with S_sel_=1 and S_gen_=0, (B) three cycles of single seed descent to S_gen_=3, and (C) five cycles of single seed descent to S_gen_=5. Size of dots is in linear proportion to 1 – population coancestry. For abbreviations, see Fig. 3 legend.

With no selfing before phenotyping (S_gen_=0), truncation selection with random or assortative mating (BlupTrunc_RAN and BlupTrunc_ASS) caused a rapid increase in index for ~15 years but then approached a plateau after 40 years. The large-high population lost most of its original genetic diversity with population coancestry 0.98 at 60 years (Fig. 5A). This reflects the tendency for truncation selection on BLUP predicted breeding values to select close relatives and lose genetic diversity over time, especially in populations with small effective population size and high selection pressure. In contrast, the index doubled in 32 years with population coancestry 0.63 in the high-index OCS option (BlupOCS_45Deg_Max5) (Fig. 5A).

Selfing to S_gen_=3 improved outcomes in the large-high population, but once again truncation selection showed major loss of genetic diversity with population coancestry 0.90 at 60 years (Fig. 5B). However, the index doubling time was reduced to 27 years and population coancestry 0.48 in the high-index OCS option (BlupOCS_45Deg_Max5). There was an advantage for conserving genetic diversity by using the high-index OCS option with maxuse=1 (BlupOCS_45Deg_Max1), with index doubling time 29 years and population coancestry 0.30.

There was no benefit from continuing single seed descent to S_gen_=5 (Fig. 5C), with doubling of index in 36 years and population coancestry 0.50 in the high-index OCS option (BlupOCS_45Deg_Max5).

### Comparison of S_0_-derived with S_3_-derived and S_5_-derived recurrent selection in the large population with moderate selection pressure

**Fig. 6.**
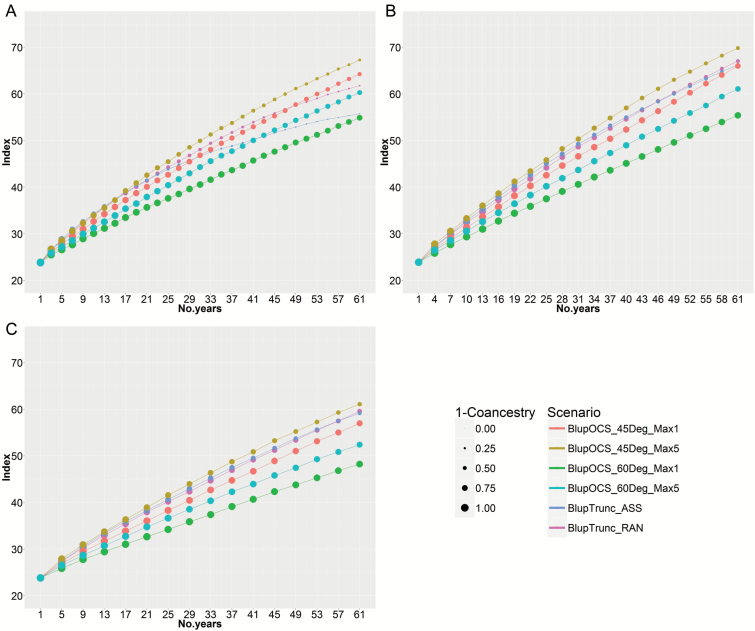
Modelling of 30 cycles of S_0_-derived recurrent selection in the large population with moderate selection pressure based on (A) the plant model with S_sel_=1 and S_gen_=0, (B) three cycles of single seed descent to S_gen_=3, and (C) five cycles of single seed descent to S_gen_=5. Size of dots is in linear proportion to 1 – population coancestry. For abbreviations, see Fig. 3 legend.

The large-moderate population produced the best results in terms of index and coancestry at 60 years. In the case of S_gen_=0, truncation selection with assortative mating (Blup Trunc_ASS) again approached a plateau with population coancestry 0.91 after 60 years (Fig. 6A). OCS, in contrast, achieved a doubling of the index in 28 years with population coancestry 0.42 in the high-index OCS option (BlupOCS_45Deg_Max5) (Fig. 6A). In the OCS solution for improved retention of genetic diversity (BlupOCS_60Deg_Max5), doubling time took 38 years with population coancestry 0.27.

Outcomes improved slightly with selfing in the large-moderate population when S_gen_=3 (Fig. 6B). The index doubled in 27 years with population coancestry 0.29 in the high-index OCS option (BlupOCS_45Deg_Max5). With truncation selection, genetic diversity was lower than with most OCS options and genetic progress was beginning to slow at 60 years (Fig. 6B).

Single seed descent to S_gen_=5 (Fig. 6C) resulted in doubling of the index in 36 years with population coancestry 0.29 in the high-index OCS option (BlupOCS_45Deg_Max5).

If single seed descent were accelerated to five generations per year, thereby reducing cycle time for S_gen_=5 from 4 to 3 years, the result for S_gen_=5 would be the same as with S_gen_=3, that is, the index would double in 27 years with population coancestry 0.29 in the high-index OCS option (BlupOCS_45Deg_Max5) (Supplementary Table S1). There was no advantage to additional generations of single seed descent.

## Discussion

We evaluated OCS for management of long-term genetic gain and genetic diversity in evolving gene banks, which are diverse pre-breeding populations of self-pollinating crops. The evolving gene bank begins by intermating elite and exotic lines, thereby moving the exotic genes from the gene bank to the field in a semi-adapted genetic background. The population is then selected for improved economic index over many cycles of recombination and selection, while retaining exotic genetic diversity for future discovery and exploitation. The methods developed here are potentially valuable for self-pollinating grain legumes, which are known for lack of genetic diversity in elite breeding programmes (Singh *et al.*, 2014). The motivating example was recurrent selection based on the animal model in field peas, a highly self-pollinating crop (Cowling *et al.*, 2015). This is the first report of the use of OCS in annual self-pollinating crops.

Most models of short-term selection response are based on the assumptions of the infinitesimal model – that each trait is controlled by an infinite number of loci each with infinitesimal and additive effects (Walsh and Lynch, 2016*c*). When effective population size was reduced from infinity to ten, response to selection ceased after 20 to 30 generations (Walsh and Lynch, 2016*c*). We also show a plateau in index after ~20 cycles in the large-high population (Fig. 5). Many crop breeding programmes have an effective population size of ten or less (Cowling, 2013) and risk reaching a plateau in genetic improvement prematurely. The aim should be to increase effective population size in evolving gene banks, so that they more closely meet the assumptions of the infinitesimal model and improve the chances of discovering and retaining new valuable exotic alleles.

In this study, OCS consistently out-performed truncation selection in terms of higher economic index and lower population coancestry in the long term (30 cycles). This is expected because BLUP-derived index values tends to rank individuals from the same pedigree closely together, which are then selected as parents, whereas OCS favours retention of genetic diversity for long-term gain by selecting parents with diverse pedigrees. S_0_-derived recurrent selection, in combination with OCS (BlupOCS_45Deg_Max5), recorded a doubling of the economic index in 28 years in the large-moderate population at which time population coancestry was 0.42 (Fig. 6A). By relaxing the emphasis in OCS on index and increasing the emphasis on lower population coancestry (BlupOCS_60Deg_Max5), the index doubled in 38 years in the large population at which time population coancestry was 0.27 (Fig. 6A).

Selfing within cycles by single seed descent to the S_3_ in the large population increased the rate of increase in index per cycle. However, the effects were marginal when taking into consideration the delay in cycle time caused by selfing (Table 3), so that doubling time was 27 years in S_3_-derived recurrent selection compared with 28 years in S_0_-derived recurrent selection (Fig. 6B, BlupOCS_45Deg_Max5). The delay in selection cycles caused by further selfing to S_5_ was not compensated by improvements in index (Fig. 6C). Limited selfing was useful but not essential for management of genetic improvement and genetic diversity in evolving gene banks. S_0_-recurrent selection may be preferred for practical reasons as 2-year cycles allow more frequent observation of material in field trials, less intensive glasshouse or laboratory work for single seed descent and therefore lower costs, and more frequent sampling of target environments than 3-year cycles. Single seed descent is useful to make pure lines from high index plants if this is important for commercial purposes.

The large population with high selection pressure (20 matings and 50 progeny per mating per cycle) achieved a poor result with truncation selection, and the economic index reached a plateau in 40 years (Fig. 5A). This was caused by the relatively low effective population size in this treatment (maximum 40 parents in matings each cycle). Clearly, it is preferable to increase effective population size by increasing the number of matings per cycle and number and diversity of parents involved in matings. This was simply achieved by changing the mating strategy to 50 matings and 20 progeny per mating per cycle in the large-moderate population, without changing the field testing budget. The small-low population suffered no plateau in economic index with truncation selection because its effective population size was large (maximum 100 parents in matings each cycle). If the budget for pre-breeding is limited, then a small population with mild selection pressure is sufficient to achieve the goals of pre-breeding in evolving gene banks, as shown here.

We included grain yield in field trials as part of the economic index. To be of practical value to elite breeding programmes in the future, evolving gene banks must undergo rapid improvement in yield and adaptation. Yield testing delays the minimum cycle time to 2 years for S_0_-derived recurrent selection, 3 years for S_3_-derived recurrent selection, and 4 years for S_5_-derived recurrent selection (Table 3).

We assumed a relatively low narrow-sense heritability for grain yield of 0.3 (Table 1). With S_0_-derived recurrent selection (2-year cycles) based on OCS for economic index, grain yield doubled to 3 t ha^-1^ in 44 years (average 2.4% per year) in the high-index OCS option (BlupOCS_45Deg_Max5) (Supplementary Table S1). For comparison, genetic improvement in grain yield of US hybrid corn doubled from 4 to 8 t ha^-1^ (average 1.8% per year) in 55 years from 1945 to 2000 (Duvick *et al.*, 2004), and grain yield of wheat in Nebraska USA increased from 3 to 4.5 t ha^-1^ (average 0.9% per year) over the same time period (Fufa *et al.*, 2005). In our modelling with OCS, evolving gene banks appear to serve their dual role of conserving genetic diversity for a wide range of traits while increasing in yield and other commercial traits.

Grain yield was only one component of the economic index. We also included black spot resistance, for which we used a narrow-sense heritability of 0.3 (Table 1) based on the results of Cowling *et al.* (2015). We predict a doubling in black spot resistance in 22–23 years with S_0_-derived recurrent selection in the high-index OCS option of the large-moderate population (BlupOCS_45Deg_Max5) (Supplementary Table S1). At the same time, grain yield improved by 60% to 2.4 t ha^-1^ and stem strength improved by 56% over starting values (Supplementary Table S1). The economic index can be adjusted to favour some traits over others (Brascamp, 1984) depending on the goal of the breeder and economic value of the trait.

In practice, valuable improvements in both yield and black spot resistance were achieved in field pea after intercrossing diverse parents followed by single seed descent to the F_5_, with disease screening on F_5_ plants, and yield and disease rating in F_6_ and F_7_ plots (Adhikari *et al.*, 2014). Our results confirm that single seed descent is not essential for long-term genetic improvement – 2-year cycles based on S_0_-derived recurrent selection with OCS for an economic index including blackspot resistance and yield achieved similar results to 3-year cycles based on S_3_-derived recurrent selection with OCS (Figs. 6A, B). With truncation selection in combination with low effective population size, as found in the large-high population, genetic improvement reached a plateau prematurely due to a loss of genetic diversity from the population (Figs. 5A, B).

The goal of pre-breeding in evolving gene banks is to conserve valuable genetic diversity derived from wild and landrace ancestors, and to improve agronomic adaptation, yield, disease resistance and other valuable traits in the population so that it becomes useful for commercial crop breeding. OCS assists the breeder in monitoring the genetic diversity in pre-breeding programmes through measurements of population coancestry. However, OCS can achieve both rapid genetic improvement *and* retention of genetic diversity in evolving gene banks more effectively than BLUP truncation selection. The strategy for selection in OCS can be changed to favor higher genetic diversity or to emphasize progeny index over population coancestry (Kinghorn and Kinghorn, 2016). Also, the weighting applied to each trait (Table 1) can be adjusted using the desired gains approach (Brascamp, 1984). If yield is considered to deserve a higher economic weighting than black spot resistance or stem strength, this can be easily changed in the economic index.

### Potential use of genomic selection in evolving gene banks

BLUP selection may be more accurate if the pedigree relationship matrix is replaced with a genomic relationship matrix (Hayes *et al.*, 2009). Similarly, a genomic relationship matrix may improve long-term genetic gain when used with OCS (Woolliams *et al.*, 2015). Genomic selection may be useful to predict breeding values of progeny for which no records exist, such as S_x+1_ candidate cross progeny, and thereby improve the efficiency of breeding in evolving gene banks. Genomic selection could be used to achieve two or three cycles of recombination on S_0_ progeny in one year, followed by retraining markers, as proposed by Rutkoski *et al.* (2011). In evolving gene banks, the retraining of markers would occur in the breeding population and not in a separate training population as proposed previously (Rutkoski *et al.*, 2011).

Genomic selection may be important to help break up large linkage blocks in evolving gene banks, and avoid the rapid reconstruction of the elite genome that occurred during pre-breeding of elite and exotic lines of maize (Gorjanc *et al.*, 2016). Valuable minor alleles from exotic sources may be discovered in evolving gene banks during genomic selection, and OCS may help the retention and exploitation of these valuable exotic alleles during pre-breeding as suggested by Gorjanc *et al.* (2016).

### The cost and returns of the evolving gene bank

The small-low population is ideal for pre-breeding in low budget situations. Both the small-low population and the large-moderate population use 50 matings per cycle and begin with crossing among 100 founder parents. Higher investment in the large-moderate population for yield testing of 1000 S_0_-derived lines is rewarded with shorter doubling time of the economic index, and higher rates of yield improvement. However, investment in large populations is wasted if selection pressure is too high and effective population size is too low, as in the large-high population with only 20 matings and 50 progeny per mating per cycle.

### Future potential use of OCS in evolving gene banks

We have compared only a small number of pre-breeding strategies to help illustrate the potential of OCS to give sustained genetic improvement. In practice, choice of strategy more appropriate to the prevailing scenario should give better results. The best strategy can be chosen in the light of the time-scale of objectives in relation to genetic improvement and management of genetic diversity. Proper implementation of OCS will ensure that maximum use of individual plants is appropriate to their genetic merit and genetic distinctiveness.

In a practical programme, other issues will need to be accommodated simultaneously with OCS, including management of known disease resistance alleles; adherence to quarantine barriers in programmes run across locations; and simultaneously handling multiple end uses, such as priming the population to more efficiently target adaptation to multiple environments or different diseases under commercial applications. Also, migration of new exotic allelic diversity into the evolving gene bank can be readily achieved with OCS, so long as effective population is high. Genetic drift tends to eliminate migrant alleles in populations with low *N*_*e*_ (Cowling, 2013).

Rapid cycle recurrent selection based on BLUP methodology with OCS is also conducive to measuring and targeting genotype by environment interaction effects in the breeding programme. High positive genetic correlations across cycles indicate low genotype by environment interaction effects, as was the case with predicted breeding values across cycles for black spot resistance in field peas (*r*=0.82) (Cowling *et al.*, 2015). However, both negative and positive genetic correlations across sites were evident for yield in genetically uniform canola lines (Beeck *et al.*, 2010; Cullis *et al.*, 2010) indicating strong genotype by environment effects for yield in that study. Wherever the environments can be classified, these can be treated as multiple end uses for simultaneous selection by OCS in the breeding programme.

The opportunities for new crop breeding methods based on BLUP-driven technologies are large, given that animal breeders have been developing these technologies since the 1960s (van der Werf, 2007) and they are only now beginning to be used in crop breeding. This paper has shown that OCS gives the control necessary to actively improve evolving gene banks for economic traits, while maintaining high levels of genetic diversity. Evolving gene banks will increase genetic diversity available to grain legume crop breeders in well-adapted and high-yielding pre-breeding pools. Evolving gene banks are a vehicle for discovery and exploitation of valuable alleles currently stored in the dormant seeds of wild and landrace grain legumes in global gene banks (Foyer *et al.*, 2016).

## Supplementary data

Supplementary data are available at JXB online.

Table S1. Mean and standard deviation (SD) from ten runs of PopSim at the end of each cycle in each selection type (SelType), population size (PopSize), mating type (Mating), S_sel_, S_gen_, maximum use as parent (Maxuse) and target degrees (Degree), of population coancestry, population inbreeding (F), Index, true breeding value (TBV) for black spot resistance (BSR), TBV for stem strength (SS), TBV for flowering time (FT) and TBV for grain yield (GY).

## Supplementary Material

supplementary_table_S1

## References

[CIT0001] Adhikari KN Khan TN Stefanova K Pritchard I 2014 Recurrent breeding method enhances the level of blackspot (*Didymella pinodes* (Berk. & Blox.) Vestergr.) resistance in ﬁeld pea (*Pisum sativum* L.) in southern Australia. Plant Breeding 133, 508–514.

[CIT0002] Allard RW 1960 Principles of plant breeding. John Wiley and Sons: New York.

[CIT0003] Beeck CP 2005 Simultaneous improvement in black spot resistance and stem strength in field pea (Pisum sativum L.). PhD Thesis. The University of Western Australia.

[CIT0004] Beeck CP Cowling WA Smith AB Cullis BR 2010 Analysis of yield and oil from a series of canola breeding trials, part I: Fitting factor analytic mixed models with pedigree information. Genome 53, 992–1001. 21076515 10.1139/G10-051

[CIT0005] Beeck CP Wroth J Cowling WA 2008 *a* Additive genetic variance for stem strength in field pea (*Pisum sativum*). Australian Journal of Agricultural Research 59, 80–85.

[CIT0006] Beeck CP Wroth JM Falk DE Khan T Cowling WA 2008 *b* Two cycles of recurrent selection lead to simultaneous improvement in black spot resistance and stem strength in field pea. Crop Science 48, 2235–2244.

[CIT0007] Bernardo R 2010 Breeding for quantitative traits in plants. 2nd ed Stemma Press: Woodbury, MN.

[CIT0008] Brascamp EW 1984 Selection indices with constraints. Animal Breeding Abstracts 52, 645–654.

[CIT0009] Cowling WA 2013 Sustainable plant breeding. Plant Breeding 132, 1–9.

[CIT0010] Cowling WA Buirchell BJ Falk DE 2009 A model for incorporating novel alleles from the primary gene pool into elite crop breeding programs while reselecting major genes for domestication or adaptation. Crop and Pasture Science 60, 1009–1015.

[CIT0011] Cowling WA Stefanova KT Beeck CP Nelson MN Hargreaves BLW Sass O Gilmour AR Siddique KHM 2015 Using the animal model to accelerate response to selection in a self‐pollinating crop. G3‐Genes Genomes Genetics 5, 1419–1428. 10.1534/g3.115.018838 PMC4502376 25943522

[CIT0012] Croser J Ahmad F Clarke H Siddique KHM 2003 Utilisation of wild *Cicer* in chickpea improvement – progress, constraints and prospects. Australian Journal of Agricultural Research 54, 429–444.

[CIT0013] Cullis BR Smith AB Beeck CP Cowling WA 2010 Analysis of yield and oil from a series of canola breeding trials, part II: Exploring variety by environment interaction using factor analysis. Genome 53, 1002–1016. 21076516 10.1139/G10-080

[CIT0014] Duvick DN Smith JSC Cooper M 2004 Long-term selection in a commercial hybrid maize breeding program. Plant Breeding Reviews 24, 109–151.

[CIT0015] Foyer CH Lam H-M Nguyen HT 2016 Neglecting legumes has compromised human health and sustainable food production. Nature Plants 2, 16112. 28221372 10.1038/nplants.2016.112

[CIT0016] Fufa H Baenziger PS Beecher BS Graybosch RA Eskridge KM Nelson LA 2005 Genetic improvement trends in agronomic performances and end-use quality characteristics among hard red winter wheat cultivars in Nebraska. Euphytica 144, 187–198.

[CIT0017] Gorjanc G Jenko J Hearne SJ Hickey JM 2016 Initiating maize pre-breeding programs using genomic selection to harness polygenic variation from landrace populations. BMC Genomics 17, 30. 26732811 10.1186/s12864-015-2345-z PMC4702314

[CIT0018] Grundy B Villanueva B Woolliams JA 1998 Dynamic selection procedures for constrained inbreeding and their consequences for pedigree development. Genetics Research 72, 159–168.

[CIT0019] Hallander J Waldmann P 2009 Optimum contribution selection in large general tree breeding populations with an application to Scots pine. Theoretical and Applied Genetics 118, 1133–1142. 19183858 10.1007/s00122-009-0968-7

[CIT0020] Harlan JR de Wet JMJ 1971 Towards a rational classification of cultivated plants. Taxon 20, 509–517.

[CIT0021] Hayes GJ Visscher PM Goddard ME 2009 Increased accuracy of artificial selection by using the realized relationship matrix. Genetics Research 91, 47–60. 19220931 10.1017/S0016672308009981

[CIT0022] Henryon M Berg P Sørensen AC 2014 Animal-breeding schemes using genomic information need breeding plans designed to maximise long-term genetic gains. Livestock Science 166, 38–47.

[CIT0023] Henryon M Ostersen T Ask B Sørensen AC Berg P 2015 Most of the long-term genetic gain from optimum-contribution selection can be realised with restrictions imposed during optimisation. Genetics Selection Evolution 47, 21. 10.1186/s12711-015-0107-7 PMC4376334 25887703

[CIT0024] Hospital F 2005 Selection in backcross programmes. Philosophical Transactions of the Royal Society B 360, 1503–1511. 10.1098/rstb.2005.1670 PMC1569518 16048792

[CIT0025] Keilwagen J Kilian B Özkan H 2014 Separating the wheat from the chaff – a strategy to utilize plant genetic resources from *ex situ* genebanks. Scientific Reports 4, 5231. 24912875 10.1038/srep05231 PMC4050481

[CIT0026] Kerr RJ McRae JA Dutkowski GW Tier B 2015 Managing the rate of increase in average co-ancestry in a rolling front tree breeding strategy. Journal of Animal Breeding and Genetics 132, 109–120. 25823837 10.1111/jbg.12157

[CIT0027] Kinghorn BP 2011 An algorithm for efficient constrained mate selection. Genetics Selection Evolution 43, 4. 10.1186/1297-9686-43-4 PMC3037843 21251244

[CIT0028] Kinghorn BP 2016 *a* GENUP. Computer aided learning for quantitative genetics. http://bkinghor.une.edu.au/genup.htm, accessed 5 October 2016.

[CIT0029] Kinghorn BP 2016 *b* DESIRE. Target your genetic gains. http://bkinghor.une.edu.au/desire.htm, accessed 5 October 2016.

[CIT0030] Kinghorn BP Banks R Gondro C Kremer VD Meszaros SA Newman S Shepherd RK Vagg RD van der Werf JHJ 2008 Strategies to exploit genetic variation while maintaining diversity. In: van der Werf JHJ Frankham R Graser H-U Gondro C, eds. Adaptation and fitness in animal populations. Springer: Berlin, 191–200.

[CIT0031] Kinghorn BP Kinghorn AJ 2016 Instructions for Matesel. http://matesel.une.edu.au, accessed 5 October 2016.

[CIT0032] Kinghorn BP Meszaros SA Vagg RD 2002 Dynamic tactical decision systems for animal breeding. Proceedings of the 7th World Congress on Genetics Applied to Livestock Production 33, 179–186.

[CIT0033] Kremer VD Newman S Wilson ER Kinghorn BP 2010 Mate Selection for sustained genetic improvement in small populations. Proceedings of the 9th World Congress on Genetics Applied to Livestock Production. Paper 0536.

[CIT0034] Lynch M Walsh B 1998 Genetics and analysis of quantitative traits. Sinauer Assoc. Inc: Sunderland, USA.

[CIT0035] Meuwissen THE 1997 Maximizing the response of selection with a predefined rate of inbreeding. Journal of Animal Science 75, 934–940. 9110204 10.2527/1997.754934x

[CIT0036] Rasmusson DC Phillips RL 1997 Plant breeding progress and genetic diversity from *de novo* variation and elevated epistasis. Crop Science 37, 303–310.

[CIT0037] Rostoks N Ramsey L MacKenzie K 2006 Recent history of artificial outcrossing facilitates whole-genome association mapping in elite inbred crop varieties. Proceedings of the National Academy of Sciences, USA 103, 18656 –18661. 10.1073/pnas.0606133103 PMC1693718 17085595

[CIT0038] Rutkoski JE Heffner EL Sorrells ME 2011 Genomic selection for durable stem rust resistance in wheat. Euphytica 179, 161–173.

[CIT0039] Siddique KHM Erskine W Hobson K Knights EJ Leonforte A Khan TN Paull JG Redden R Materne M 2013 Cool-season grain legume improvement in Australia — use of genetic resources. Crop and Pasture Science 64, 347–360.

[CIT0040] Singh M Bisht IS Dutta M 2014 Broadening the genetic base of grain legumes. Springer India: New Delhi.

[CIT0041] Tanksley SD Nelson JC 1996 Advanced backcross QTL analysis: a method for the simultaneous discovery and transfer of valuable QTLs from unadapted germplasm into elite breeding lines. Theoretical and Applied Genetics 92, 191–203. 24166168 10.1007/BF00223376

[CIT0042] Tester M Langridge P 2010 Breeding technologies to increase crop production in a changing world. Science 327, 818–822. 20150489 10.1126/science.1183700

[CIT0043] van der Werf J 2007 Animal breeding and the black box of biology. Journal of Animal Breeding and Genetics 124, 101. 17550349 10.1111/j.1439-0388.2007.00657.x

[CIT0044] Walsh B Lynch M 2016 *a* Analysis of short-term selection experiments: 2. Mixed-model and Bayesian approaches. Chapter 19 In: Evolution and selection of quantitative traits: I. Foundations http://nitro.biosci.arizona.edu/zbook/NewVolume_2/pdf/WLChapter19.pdf, accessed 5 October, 2016.

[CIT0045] Walsh B Lynch M 2016 *b* Selection under inbreeding. Chapter 23 In: Evolution and selection of quantitative traits:I. Foundations http://nitro.biosci.arizona.edu/zbook/NewVolume_2/pdf/WLChapter23.pdf, accessed 5 October, 2016.

[CIT0046] Walsh B Lynch M 2012 *c* The inﬁnitesimal model and its extensions. Chapter 24 In: Evolution and selection of quantitative traits: I. Foundations http://nitro.biosci.arizona.edu/zbook/NewVolume_2/pdf/Chapter24.html, accessed 5 October 2016.

[CIT0047] Webb SL Demarais S Strickland BK DeYoung RW Kinghorn BP Gee KL 2012 Effects of selective harvest on antler size in white-tailed deer: a modeling approach. Journal of Wildlife Management 76, 48–56.

[CIT0048] Woolliams JA Berg P Dagnachew BS Meuwissen THE 2015 Genetic contributions and their optimization. Journal of Animal Breeding and Genetics 132, 89–99. 25823835 10.1111/jbg.12148

[CIT0049] Zamir D 2001 Improving plant breeding with exotic genetic libraries. Nature Reviews Genetics 2, 983–989. 10.1038/35103590 11733751

